# Septic Shock After Intravesical Therapy With Bacillus Calmette-Guerin: A Case Report of a Rare Life-Threatening Complication

**DOI:** 10.7759/cureus.46563

**Published:** 2023-10-06

**Authors:** Pedro Francisco Fernandes, Pedro Nunes, Arnaldo Figueiredo

**Affiliations:** 1 Department of Urology and Renal Transplantation, Centro Hospitalar e Universitário de Coimbra, Coimbra, PRT

**Keywords:** bacillus calmette-guerin, systemic infection, side effects, intravesical therapy, bladder cancer

## Abstract

Intravesical therapy with Bacillus Calmette-Guerin (BCG) is the mainstay treatment for high-risk non-muscle invasive bladder cancer. The side effects are usually local and mild. Systemic dissemination of BCG is rare, typically develops soon after instillation, and may present as a severe life-threatening condition.

We present a case of a 49-year-old man under chronic haemodialysis who developed septic shock after the first BCG maintenance instillation for bladder carcinoma *in situ* (CIS). Supportive measures and empiric broad-spectrum antibiotic therapy were promptly started after sample collection for cultures. Lastly, the recurrence of fever raised the initial suspicion of BCG dissemination. The diagnosis was confirmed by the identification of the *Mycobacterium tuberculosis *complex in blood samples collected and anti-tuberculosis therapy was then initiated.

We would like to highlight the need for early recognition of a systemic BCG infection and the importance of starting anti-tuberculosis treatment as early as possible.

## Introduction

Intravesical Bacillus Calmette-Guerin (BCG) is established as an effective treatment to prevent the recurrence and progression of intermediate and high-risk non-muscle invasive bladder cancer [1]. BCG is a lived attenuated strain of *Mycobacterium bovis* which induces a cell-mediated immune response [2,3]. Although usually safe and well tolerated, the treatment may cause some side events. These are mainly mild and limited to the genitourinary tract and include storage bladder symptoms (35.0%), bacterial cystitis not BCG-related (23.3%), macroscopic haematuria (22.6%) [4], and epididymo-orchitis (up to 3.5%) [2,5,6] or symptomatic granulomatous prostatitis (up to 1.0%) [4,7,8]. Systemic side effects such as general malaise, myalgia, nausea, chills, and low-grade fever usually resolve spontaneously within 48 hours [2,4-6]. However, careful monitoring and diagnostic workup is required in patients with high-grade fever that last 12 to 48 hours [5,6]. In addition to local complications, its presence may indicate less frequent systemic adverse events such as pneumonitis (0.7%) [5] or hepatitis (up to 5.7%) [5,6]. Disseminated BCG infection presenting as sepsis is an unusual complication, occurring in less than 0.4% of patients [4,5].

Also called BCGitis, the disseminated BCG infection should be suspected in any patient who develops moderate to severe genitourinary or systemic symptoms after intravesical instillation, when an alternative diagnosis is unlikely [9]. The treatment requires anti-tuberculosis drugs [1,3,4,6,7,10-12].

We present a case of disseminated *Mycobacterium bovis* infection that presented as a septic shock following intravesical therapy with BCG.

## Case presentation

**Figure 1:**
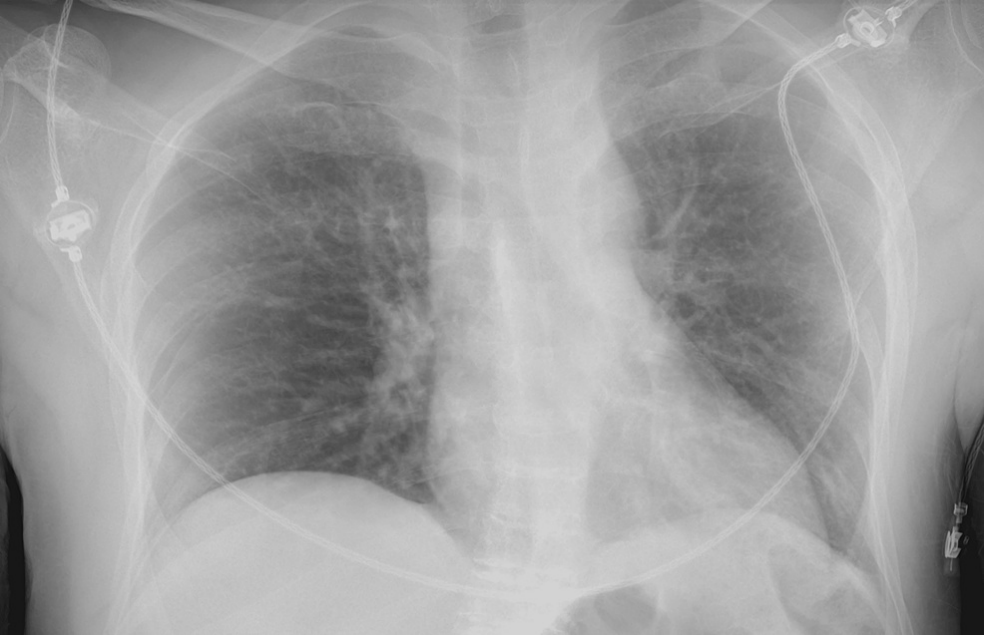
Chest radiograph - anteroposterior view.

**Figure 2:**
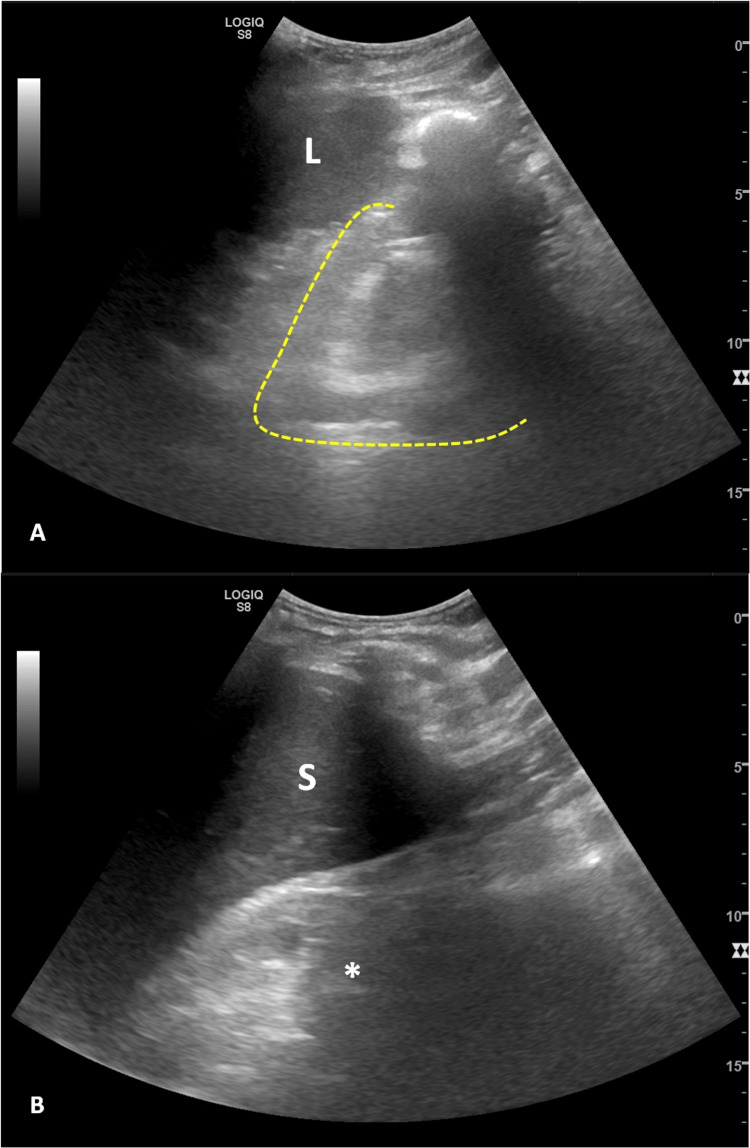
Abdominal and renal ultrasound without apparent abdominal changes and demonstrating a marked reduction in the differentiation of the right kidney (A - yellow dashed line) and absence of the left kidney (B - asterisk). Study limited by gas interposition. L, liver; S, spleen.

A 49-years-old man with stage 5 chronic kidney disease on regular haemodialysis programme and with low diuresis was diagnosed with superficial urothelial bladder cancer (pTa, high grade, G2) after a transurethral resection of bladder tumour (TURBT) and submitted to intravesical chemotherapy with mitomycin C. One year after, high-grade urothelial carcinoma was identified in bladder-washer cytology and a systematic bladder biopsy revealed carcinoma *in situ* (CIS). Treatment with BCG was started and the patient finished the induction without any complications. One day after the first maintenance instillation patient presented to the emergency department due to asthenia and fever. He reported having had haematuria and was lethargic and hypotensive with signs of poor peripheral perfusion (cold extremities and prolonged capillary refill time), with the tympanic temperature being 36.7ºC. Fluid challenge and empirical antibiotic therapy with piperacillin/tazobactam and vancomycin were promptly started after collecting urine and blood samples to culture including in BACTEC^TM^ system (Becton, Dickinson and Company, Franklin Lakes, USA). Due to refractory arterial hypotension, sympathomimetic amine support was started. Admission blood tests showed markedly increased inflammatory parameters (C-reactive protein 18.1 mg/dL, reference range < 0.5; procalcitonin 353 ng/mL, reference range < 0.5) as well as liver dysfunction (total bilirubin 2.0 g/dL, range 0.2 - 1.2; alkaline phosphatase 357 U/L, range 30 - 120; gamma-glutamyl transferase 766 U/L, range < 55; alanine aminotransferase (ALT) 908 U/L, range < 35; aspartate aminotransferase (AST) 502 U/L, range < 45 and lactate dehydrogenase (LDH) 502 U/L, range < 248) (Table 1). Chest radiograph and abdominal ultrasound were unremarkable (Figures 1, 2).

**Table 1:** Laboratory values upon presentation.

Parameter	Result	Reference range
Haemoglobin	12.0 g/dL	12.0 - 15.6
Leucocytes	17.9 x10^9^/L	3.90 - 10.2
Neutrophils	17.41 x10^9^/L	1.50 - 7.70
Platelets	155 x 10^9^/L	150 - 450
Urea nitrogen	120.0 mg/dL	7.9 - 20.9
Na^+^	133 mmol/L	136 - 146
K^+^	5.8 mmol/L	3.5 - 5.1
Cl^-^	96 mmol/L	101 - 109
Total bilirubin	2.0 mg/dL	0.2 - 1.2
Gamma-glutamyl transferase	766 U/L	< 55
Alkaline phosphatase	357 U/L	30 - 120
Alanine aminotransferase	908 U/L	< 35
Aspartate aminotransferase	502 U/L	< 45
Lactate dehydrogenase	608 U/L	< 248
Protein C reactive	18.1 mg/dL	< 0.50
Procalcitonin	353 ng/mL	0 - 0.5

During the first week of hospitalization, the patient presented good clinical evolution and concomitant decrease in inflammatory parameters, with a progressive reduction in noradrenaline needs, which was discontinued six days later. By that time no microorganism had been identified in cultures. On the sixth day of hospitalization, the patient was diagnosed with asymptomatic SARS-CoV-2 infection and was isolated for 7 days. Two weeks after the admission, there was a recurrence of fever, which persisted mainly in the afternoon. On the 23rd inpatient day, the laboratory notified us about the preliminary isolation and identification of the *Mycobacterium tuberculosis* complex in blood cultures collected at admission. Empirical treatment for tuberculosis was started with isoniazid 300 mg once a day (od), rifampicin 600 mg od, pyrazinamide 1500 mg, ethambutol 900 mg and pyridoxine 75 mg, the last three drugs taken after each session of haemodialysis. Given the subsequent sustained apyrexia, the patient was discharged ten days later and referred to the Pneumology outpatient clinic to proceed with the treatment. Sixty days after the isolation of the microorganism, resistance to pyrazinamide was notified and ultimately *Mycobacterium bovis* was identified. The patient proceeded with the treatment with isoniazid 300 mg od, rifampicin 600 mg od and pyridoxine 75 mg after haemodialysis sessions for seven months. There were no side effects reported and the patient is currently asymptomatic and with no signs of active infection. 

Regarding the neoplastic disease, the patient underwent a systematic bladder biopsy which has put in evidence the absence of CIS. Six months later, the patient is still under surveillance, being submitted to cystoscopy and urinary cytology every three months which haven't shown signs of recurrence.

## Discussion

It is not completely understood the mechanism by which BCG leads to the development of systemic disease, whether a hypersensitivity reaction type 4 or an ongoing dissemination of *Mycobacterium bovis* in the involved tissue [7,9,12]. The presence of granulomas in the absence of positive staining for acid-fast bacilli, culture and polymerase chain reaction testing for mycobacterial DNA and the resolution of symptoms that only occur after adding corticosteroids favour the inflammation hypothesis [7,9,10,12]. On the other hand, the presence of viable organisms in a variety of tissues supports the haematogenous theory [12]. Possibly both mechanisms may contribute to the pathogenesis of distant complications [12]. Indeed, disruptions of urothelium may trigger the haematogenous spread of *Mycobacterium bovis* [8,11,13]. The risk is increased subsequently to traumatic catheterization or recent TURBT [1,8,11], in the presence of macroscopic haematuria and/or urinary tract infection [1,11]. At this point, we are convinced that the dissemination of *Mycobacterium bovis* in our case has originated in a traumatic catheterization associated with the administration of BCG.

Although data are still lacking, the presence of underlying immunosuppression [6,14] or low or no residual diuresis, both present in our patient, are also considered potential risk factors for local and systemic complications, due to a longer stay of BCG in the bladder [14]. Nonetheless, reduced doses don’t look to decrease toxicity and long-duration protocols do not lead to increased toxicity [4]. In fact, the majority of side effects occur within the first year of treatment which suggests they are mainly dependent on the host and not on the number of installations [4].

Persistent high-grade fever that lasts 12 to 48 hours after intravesical instillation and is accompanied by other systemic manifestations should promptly raise the suspicion of dissemination of BCG [4-6,12]. Haematuria during the course of treatments, as in the case reported, reinforces this diagnosis, and the presence of organ dysfunction highlights a severe condition.

Of note, there is no reliable laboratory test available to confirm BCG systemic infection. Nonetheless, and despite their low yield [3,4], cultures of blood and urine should be performed [3]. Sputum and bronchoalveolar lavage should be obtained if there are respiratory symptoms [6]. Chest radiography, or preferably computerized tomography scan, should be done in the presence of systemic symptoms to rule out miliary involvement [6]. In fact, the presumptive diagnosis of systemic dissemination of BCG has just been made after the identification of the *Mycobacterium tuberculosis *complex on blood samples collected on admission, which consequently has delayed the initiation of proper management. The resistance of the microorganism to pyrazinamide strengthened our diagnostic hypothesis [15], which was later confirmed with the identification of the species.

The European Association of Urology and the European Organisation for Research and Treatment of Cancer recommend cessation of BCG and starting antituberculosis treatment with isoniazid 300 mg, rifampicin 600 mg, and ethambutol 1200 mg daily for 6 months [1,4]. Alternatively, high-dose quinolones could be used [1]. High-dose corticosteroids may also be indicated [1,4], although in our case they were not used.

In most patients, the prognosis is favourable when appropriate therapy is started. The mortality attributable to BCG infection is 5.4%, achieving 9.9% in patients with disseminated disease [6].

## Conclusions

BCG immunotherapy is safe and usually produces only mild side effects. However, severe systemic manifestations may occur. Therefore, a low threshold of suspicion for this clinical entity is mandatory. In the setting of serious disseminated disease, a trial of anti-mycobacterial therapy should be started. Of note, negative cultures do not exclude the diagnosis.
